# Trajectories of Symbolic and Nonsymbolic Magnitude Processing in the First Year of Formal Schooling

**DOI:** 10.1371/journal.pone.0149863

**Published:** 2016-03-01

**Authors:** Anna A. Matejko, Daniel Ansari

**Affiliations:** Numerical Cognition Laboratory, Department of Psychology and Brain & Mind Institute, Westminster Hall, Western University, London ON, Canada; University of Padova, ITALY

## Abstract

Sensitivity to numerical magnitudes is thought to provide a foundation for higher-level mathematical skills such as calculation. It is still unclear how symbolic (e.g. Arabic digits) and nonsymbolic (e.g. Dots) magnitude systems develop and how the two formats relate to one another. Some theories propose that children learn the meaning of symbolic numbers by scaffolding them onto a pre-existing nonsymbolic system (Approximate Number System). Others suggest that symbolic and nonsymbolic magnitudes have distinct and non-overlapping representations. In the present study, we examine the developmental trajectories of symbolic and nonsymbolic magnitude processing skills and how they relate to each other in the first year of formal schooling when children are becoming more fluent with symbolic numbers. Thirty Grade 1 children completed symbolic and nonsymbolic magnitude processing tasks at three time points in Grade 1. We found that symbolic and nonsymbolic magnitude processing skills had distinct developmental trajectories, where symbolic magnitude processing was characterized by greater gains than nonsymbolic skills over the one-year period in Grade 1. We further found that the development of the two formats only related to one another in the first half of the school year where symbolic magnitude processing skills influenced later nonsymbolic skills. These findings indicate that symbolic and nonsymbolic abilities have different developmental trajectories and that the development of symbolic abilities is not strongly linked to nonsymbolic representations by Grade 1. These findings also suggest that the relationship between symbolic and nonsymbolic processing is not as unidirectional as previously thought.

## Introduction

Early numeracy skills are thought to set the foundation for later developing mathematical skills such as arithmetic [1,2]. In order to continue on to more formal mathematics, children first need to learn what number symbols (Arabic digits) mean and have an understanding of the magnitudes they represent (e.g. knowing that the Arabic digit 4 represents four discrete items). Sensitivity to numerical magnitudes is thought to be an especially important numerical competency, particularly in the first years of schooling [2–6]. Numerical magnitudes can be represented both symbolically (e.g. Arabic digits) and non-symbolically (e.g. dots). How children learn the meaning of number symbols is still poorly understood and several questions remain unanswered about the development of symbolic and nonsymbolic number representations. Namely, how do these representations change over developmental time, and are they underlain by the same or distinct underlying mechanisms?

### Do symbolic and nonsymbolic numbers have the same underlying representation?

A large body of evidence has accumulated to show that infants have a rudimentary number sense allowing them to discriminate between two sets of dots (nonsymbolic representations of number) as long as the difference between the two sets is sufficiently large [7]. Infants are able to compare and discriminate between quantities irrespective of the physical properties of the dots (i.e. surface area, density, contour length etc.), suggesting that they are specifically tuned to the numerical magnitudes [8]. This ability also appears to be shared with non-human primates who have been shown to discriminate between numerical magnitudes [9]. Infants and nonhuman primates are therefore thought to have nonsymbolic representations of number. These rudimentary nonsymbolic representations of number have both phylogenetic and ontogenetic continuity [9]. This ability to rapidly and approximately estimate and compare nonsymbolic numerical quantities is also known as the Approximate Number System (ANS) [10].

A hallmark characteristic of the ANS is that numerical magnitude representations are thought to become more imprecise (and noisy) with increasing magnitude [11]. Consistent with this model of magnitude representation, it has been shown that when children, adults, or non-human primates compare the relative magnitude of two symbolic or nonsymbolic numbers, the speed and accuracy of comparisons is related to the ratio between the numbers (smaller number/larger number). More specifically, reaction times and errors increase as the ratio between the two numbers increases. Larger ratios are thought to be more difficult to compare due to a higher degree of overlap in the representations for the numbers, in contrast, smaller ratio pairs have less representational overlap making it easier to compare the quantities. As children get older, they become faster and more accurate at comparing relative quantities, which is thought to reflect changes in their internal number representations [12,13].

Against the background of data showing that nonsymbolic numerical magnitude processing is similar in infants, children, adults, and non-human animals, it has been suggested that early nonsymbolic representations provide the basis from which symbolic numbers (Arabic digits, such as 2 or 5) are learned. Specifically, it has been argued that over the course of learning and development number symbols become attached to their nonsymbolic quantity representations [10,14,15]. This theory suggests that symbolic number representations, which are uniquely human and culturally dependent, could be mapped onto a more evolutionarily ancient nonsymbolic system (ANS). The existing literature posits that symbolic and nonsymbolic representations become integrated into one representational system in older children and adults [16]. In other words, number symbols become linked to the ANS (or the nonsymbolic system) through a process of mapping number symbols onto their corresponding nonsymbolic quantities, particularly between ages 6–8 [17]. Critically, children’s mapping abilities have been associated with individual differences in math achievement [16–18], suggesting an important role for mapping in the development of symbolic representations and math development.

Several pieces of evidence have been put forward in support of the notion that symbolic number representations are scaffolded onto a nonsymbolic system and share the same underlying representation. As discussed above, both number symbols and nonsymbolic magnitude comparisons elicit the characteristic numerical ratio effect, which is evident early in development [10,19]. Symbols are thought to ‘inherit’ the ratio-dependent properties of nonsymbolic quantities when they are learned. Research demonstrating a relation between symbolic and nonsymbolic number processing in kindergarten or younger children (such as mapping between symbolic and nonsymbolic formats, discussed above) has also been used as evidence to support the theory that symbolic number abilities are scaffolded onto a nonsymbolic system [17]. Additional evidence has also suggested that individual differences in the ANS predict symbolic mathematical skills [20–22], which is thought to reflect a link between earlier nonsymbolic processing and formal mathematics. Finally, brain imaging evidence pointing to similar neural substrates for symbolic and nonsymbolic numbers has also been cited as potential evidence that the two systems have one common underlying representation [23,24].

Recently, however, a growing body of evidence has begun to question the strong relationship between nonsymbolic and symbolic representations. Several longitudinal studies have not found evidence to support the notion that symbolic representations are mapped on to the ANS (discussed below) [25,26]. The validity of symbolic ratio effects have also recently been questioned by Lyons, Neurk and Ansari [27] who demonstrated that only 30% of children had a reliable symbolic ratio effect even when 75% of those children had a reliable nonsymbolic ratio effect. Moreover, whether a child had a significant nonsymbolic ratio effect did not predict a significant symbolic ratio effect, suggesting that there may be no common underlying representation. The reliability of the link between nonsymbolic performance and mathematics has also been subject to some debate because many studies have not found such a relationship [2]. Finally, while some evidence suggests common underlying brain mechanisms for symbolic and nonsymbolic processing, recent evidence suggests that the underlying neural representations for the two formats are fundamentally different [28,29]. Together, these findings present a mixed picture, with some research supporting a common underlying mechanism for symbolic and nonsymbolic processing and other research that does not. Therefore, there is not unequivocal support for the theory that numerical symbols become representations of numerical magnitude by being mapped onto the pre-existing system for the approximate representation of nonsymbolic numerical magnitude.

### Two distinct representations of symbolic and nonsymbolic numbers

The idea of symbols being scaffolded on to a nonsymbolic system provides a compelling hypothesis, however, recent evidence has contested the idea that symbolic and nonsymbolic numbers have a shared representation. In a study with adults, Lyons, Ansari, and Beilock [30] provided evidence to suggest that symbolic and nonsymbolic representations may be more distinct that previously assumed. Specifically, these authors found that adults find comparison across formats significantly harder than comparison of representations within a format (e.g. symbolic-symbolic). If, as suggested by the dominant theories, numerical symbols are intrinsically tied to the nonsymbolic magnitudes they represent, then performance on a mixed format number comparison task (i.e. determining whether an array of dots or an Arabic numeral is larger) should not be associated with any processing costs. Contrary to this prediction, Lyons et al. [30] found that adults had significantly poorer performance when they were asked to compare across symbolic and nonsymbolic formats than when they were asked to compare two nonsymbolic quantities. The processing cost in the symbolic and nonsymbolic comparison did not appear to be associated with mixing visual formats because there were no decrements to performance when two different symbolic formats were compared (numerals and number words).

There is also developmental evidence to suggest that nonsymbolic representations of magnitude are not the foundation on which symbolic numbers are learned. For example, if symbolic representations were mapped on to approximate numerical quantities, one would expect that individual differences in ANS acuity would predict fluency with symbolic numbers as children learn to acquire the meaning of number symbols. In a recent study, this prediction was not supported by the data. Specifically, Sasanguie et al. [25] demonstrated that performance on a nonsymbolic task in kindergarten did not predict symbolic number processing 6 months later. Similarly, Mussolin et al. [26] found that symbolic number knowledge and cardinal number knowledge (knowing the number word for the number of items in a set) predicted ANS acuity 7 months later, however, the reverse was not found to be true; ANS acuity did not predict the acquisition of symbolic and cardinal number knowledge. These findings indicate that the early acquisition of symbolic numbers may help refine and tune nonsymbolic representations and that there is a directional association between symbolic number knowledge and the ANS, but it is not necessarily in the direction that would be predicted from the mapping hypothesis. Crucially, this literature does not question whether or not the ANS exists, but rather it questions whether symbols are grounded in this nonsymbolic representational system. The research summarized above, along with a growing body of cross-sectional literature that demonstrates weak or non-significant correlations between symbolic and nonsymbolic tasks, casts some doubt on whether symbolic and nonsymbolic numbers share the same representation [31–33].

### Rationale for the present study

The literature discussed above presents a mixed picture of the relationship between symbolic and nonsymbolic number representations in young children. In particular, it is still unclear how symbolic and nonsymbolic number representations develop over time, and whether they are more closely linked earlier in development. One could posit that younger children should have greater fluency with nonsymbolic quantities because nonsymbolic number processing exists from infancy onwards. In contrast, the meanings of symbolic numbers need to be learned [34]; as children become more fluent with symbolic numbers, they may show increasing proficiency on symbolic number comparison tasks. Indeed, in a recent study children in Grade 1 (6.7 years old) were found have better performance on a nonsymbolic task compared to a symbolic comparison task, whereas by Grades 2 and 3, children (7.7 and 8.7 years old, respectively) were found to be performing equally well in both formats [35]. These results indicate that Grade 1 may be a particularly important period for the development of symbolic numbers. Importantly, it may also suggest younger children have strong pre-existing nonsymbolic number representations from which symbolic representations acquire their meaning.

Against this background, the present longitudinal study aims to examine the nature of the relationship between symbolic and nonsymbolic number processing in the first year of formal schooling. Previous cross-sectional research has suggested that Grade 1 may be a particularly important period for fluency with symbolic numbers [35]. Therefore, we aim to investigate how symbolic and nonsymbolic number representations change across Grade 1 using a longitudinal design.

More specifically, the present study examines two primary questions related to the development of symbolic and nonsymbolic number processing in the first grade: 1) how these two representations develop within the first year of formal schooling as children are becoming more fluent with symbolic numbers and 2) whether development in one system is related to improvements in the other. If symbolic representations were scaffolded on nonsymbolic representations, we would expect that performance on the symbolic comparison would lag behind the nonsymbolic task at the beginning of Grade 1. Crucially, if symbolic and nonsymbolic magnitudes rely on the same underlying representation, then individual differences in performance on the nonsymbolic task should be highly predictive of performance on the symbolic task throughout the year. We would also expect that changes in the nonsymbolic system would correlate with improvements in the symbolic system.

## Method

### Participants

The procedures and materials of the present study were approved by the Health Sciences Research Ethics Board of the University of Western Ontario. Written consent for participation in the study was obtained from the next of kin, caretakers, or guardian on behalf of the children enrolled in the study. Thirty-one typically developing Grade 1 students were recruited to participate in a longitudinal study on the development of numerical and mathematical skills. There was no attrition across the three testing points and the 31 children completed all sessions. One child was excluded from the analyses due to poor accuracy (fewer than 50% correct on attempted items) on the symbolic task at the first time point, resulting in a final sample of 30 children (16 female). Children were an average of 6.35 years old (SD = 0.21 years) at the first testing session.

### Tests and Materials

#### Procedure

Paper-and-pencil symbolic and nonsymbolic magnitude comparison tasks were administered to children at the beginning (Time 1: September-October), middle (Time 2: January-February), and end of the school year (Time 3: May-June). The average time between sessions was 3.76 months between Time 1 and Time 2 (Range = 3.23–4.51 months), and 4.12 months between Time 2 and Time 3 (Range = 3.61–4.63 months). The magnitude comparison tasks were part of a bigger battery of math and reading tests, and the magnitude comparison task was administered in the middle of the testing session. Children were individually tested in a quiet room at the university with a trained examiner.

There were two task orders where the symbolic task was presented first in Order A and the nonsymbolic task was presented first in Order B. The order of tasks were counterbalanced across children where half the participants (16 children) received Order A at Time 1, Order B at Time 2, and Order A at Time 3. The other children were presented with the opposite order (Order B, Order A, Order B). Consequently, at each testing session half the children were first presented the symbolic task and the other half were presented the nonsymbolic task first.

#### Magnitude Comparison Task

Symbolic and nonsymbolic magnitude processing skills were assessed using a paper-and pencil magnitude comparison task. The same task has previously been used with children from Kindergarten to Grade 3 [35,36] and has proved to be a reliable and valid measure of children’s magnitude processing skills (See [33] and www.numeracyscreener.org). Children’s performance on this task has also been shown to correlate with individual differences in math achievement [35].

Participants were presented with a booklet of symbolic (Arabic digits) and nonsymbolic (dots) number pairs and were asked to compare two numerical magnitudes and strike through (using a pencil) the larger number. Magnitudes ranged from 1–9 and the side on which the larger magnitude was presented was counterbalanced across items. For each format, children were presented with 56 items (56 symbolic pairs and 56 nonsymbolic pairs). Within each format, items increased in difficulty by manipulating the numerical ratio between the two numbers. Easier items were presented earlier in the test using smaller ratios pairs (such as 3 vs 9, a ratio of .33), and items progressively got harder by increasing the ratio between the digits (such as 8 vs 9, a ratio of .89). Ratios ranged from .11 to .89 (see [33] for the number pairs and ratios used in the task). The trial order was slightly varied for the symbolic and nonsymbolic tasks to ensure the order of presentation was not identical for both tasks. To help ensure that children were not using non-numerical cues to solve the nonsymbolic task, the two dot arrays were equated on cumulative surface area in half the trials, and total perimeter in the other half. When the arrays were matched on total area, the larger array had a greater cumulative perimeter. When the arrays were matched on total perimeter, the larger array occupied a greater area. Area and perimeter matched trials were randomly presented through the task so that children could not anticipate which perceptual cues varied with numerical size. Due to the nature of nonsymbolic stimuli, it is not possible to perfectly control for non-numerical cues [37,38]. With some controls over non-numerical parameters it makes it difficult for children to do the task using perceptual cues alone. However, as with all nonsymbolic tasks, it is not possible to isolate nonsymbolic numerical processing from the influence of continuous parameters [38]. Consequently, performance on the nonsymbolic task could reflect ANS acuity, the ability to detect non-numerical continuous cues, or a combination of the two. Whereas performance on the symbolic comparison task is arguably less influenced by non-numerical cues and could be a more process-pure measure (for a greater discussion on these factors please see [38]).

To familiarize children with the task, they were first presented with 3 sample items that the experimenter did together with the child, followed by 9 practice items completed by the child. These practice items ensured that children were familiar with digits from 1–9 and understood the task. Children were given feedback on the practice items if necessary. Sample and practice items were presented before both symbolic and nonsymbolic formats. Children were instructed to strike through the larger number, and if they made a mistake they were told to cross out the item they did not want and strike through the correct side. Children were then given 1 minute to complete as many items as possible for each format (2 minutes in total for both symbolic and nonsymbolic formats). Consequently, raw scores (number of total correct items) on the task reflect both accuracy and speed.

Of the 56 items on the symbolic task, children attempted an average of 28.8 (T_1_ Range: 21–39), 33.1 (T_2_ Range: 17–43), and 37.5 (T_3_ Range: 28–50) items. Of the 56 items on the nonsymbolic task, children attempted an average of 32.7 (T_1_ Range: 21–43), 36.7 (T_2_ Range: 22–53), and 39.1 (T_3_ Range: 28–56) items. Out of the total number of attempted items by each child, children were highly accurate on the symbolic (T_1_ = 99.5%; T_2_ = 99.6%; T_3_ = 99.1%) and nonsymbolic tasks (T_1_ = 96.8%; T_2_ = 95.8%; T_3_ = 97.0%), indicating that they understood the instructions. All further analyses were performed on the raw scores (total number of correct items).

## Results

### Development of symbolic and nonsymbolic magnitude processing skills

**Fig 1:**
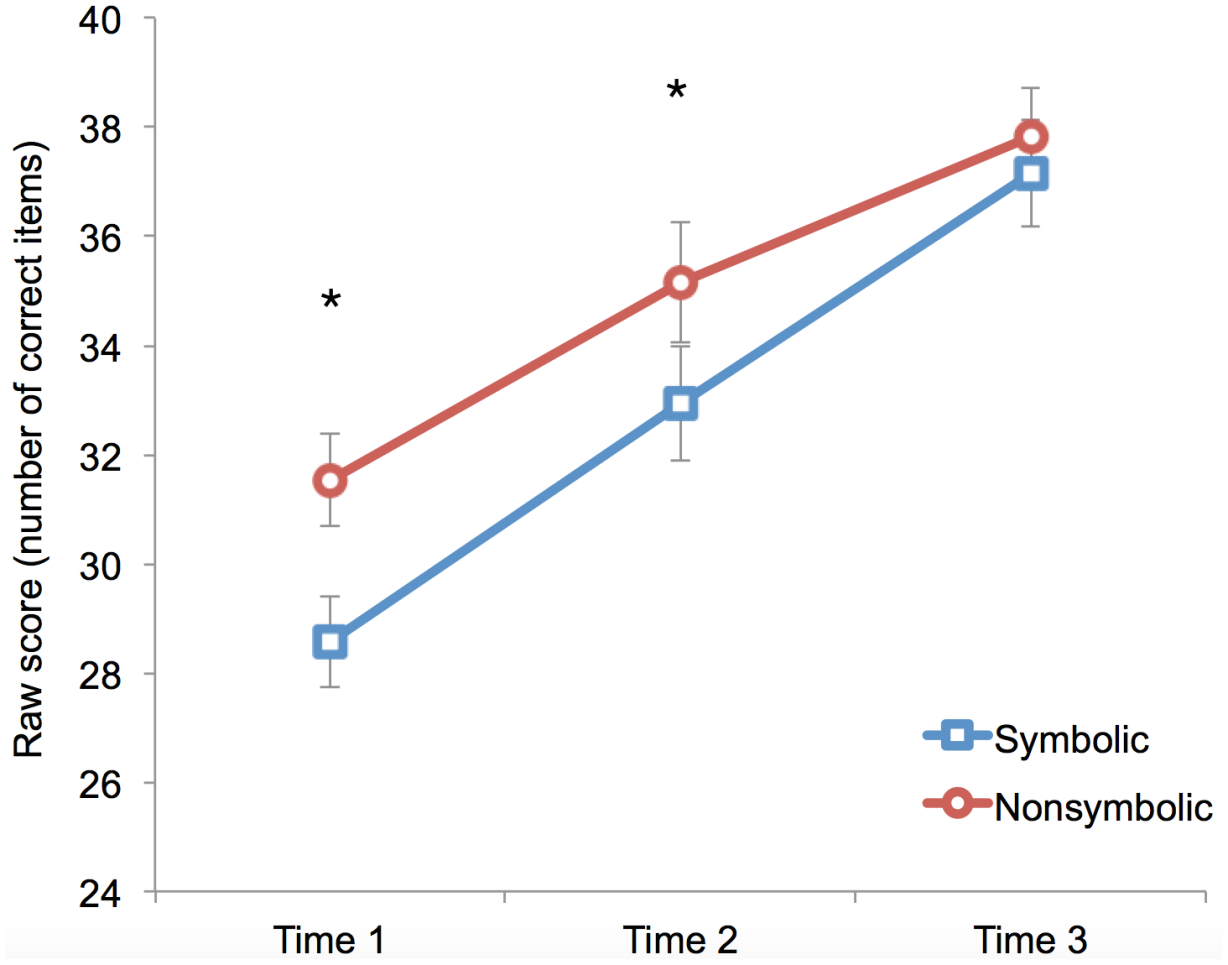
The development of symbolic and nonsymbolic skills in Grade 1.

To examine how symbolic and nonsymbolic magnitude processing skills change over the first year of formal schooling we conducted a within-subjects ANOVA with task (symbolic, nonsymbolic) and time (Time 1, Time 2, Time 3) as within subject factors. This analysis revealed a main effect of Task F(1, 29) = 13.28, *p* = .001, η^2^ = .06, a main effect of Time, F (2, 58) = 77.41, *p* < .001, η^2^ = .53, and a Task x Time interaction F(2, 58) = 4.47, *p* = .006, η^2^ = .01. As can be seen in Fig 1, children performed better on the nonsymbolic task (M = 34.9) than the symbolic task (M = 32.9), and performed better over time (M_T1_ = 30.1, M_T2_ = 34.1, M_T3_ = 37.5). Bonferroni corrected post-hoc tests were conducted to further examine the Task x Time interaction. Children performed significantly better on the nonsymbolic task at Time 1 (*p* < .001) and Time 2 (*p* = .002), but performed equally well on the tasks at Time 3 (*p* = .33). This indicates that although nonsymbolic performance is higher than symbolic performance at the beginning of the school year, children perform equally well on the two tasks by the end of the school year.

We also investigated whether performance on the two tasks becomes more similar over time by conducting correlations between symbolic and nonsymbolic performance at each time point. Symbolic and nonsymbolic performance was significantly correlated at Time 1 (r(28) = .60, *p* < .001), at Time 2 (r(28) = .83, *p* < .001), and Time 3 (r(28) = .72, *p* < .001). Using Steiger’s Z-test to compare two dependent correlations [39,40], we found that the correlation between symbolic and nonsymbolic performance significantly increased from Time 1 to Time 2 (Steiger’s Z = -2.23, *p* = .001), but not from Time 2 to Time 3 (Steiger’s Z = 1.26, *p* = .21).

### Changes in performance over time

In order to estimate individual differences in changes in performance over time, we estimated linear slopes for each individual for the symbolic and nonsymbolic tasks. Because there was some inter-subject variability in the time between testing sessions (see Procedure above), we accounted for the number of months between sessions when calculating the slopes (changes in the value of ‘x’ were a function of the number of months from Time 1). Individual slopes for the symbolic and nonsymbolic tasks were then used as an indicator of the degree of change, or growth, over time. The slopes for the symbolic and nonsymbolic tasks both significantly differed from zero, t(29) = 12.65, *p* < .001 and t(29) = 10.161, *p* < .001, respectively. Furthermore, a linear function fit changes in both symbolic (R^2^ = .299) and nonsymbolic (R^2^ = .192) performance. To test whether change in performance over time was different between the symbolic and nonsymbolic tasks, a paired-samples t-test was used to compare the average slope. The slope for the symbolic task (M = 1.09) was significantly greater than the nonsymbolic task (M = .81), t(29) = 2.82, *p* = .008, suggesting a greater increase in symbolic performance over time.

To investigate whether changes in performance on the symbolic task were related to changes in the nonsymbolic task, we correlated slopes for the symbolic and nonsymbolic tasks. We found that improvements on the nonsymbolic task did not significantly correlate with improvements on the symbolic task r(28) = .28, *p* = .13, suggesting that gains in one format did not correspond with gains in the other. Because the relationship between symbolic and nonsymbolic trajectories may change over the year, we further examined how changes in symbolic and nonsymbolic performance relate to one another in the first half of the year and in the second half of the year. To investigate this, we calculated change scores (e.g. T2-T1) while controlling for the individual differences in the time between sessions (e.g. [Raw scores T2- Raw scores T1] / [Age in months T2 –Age in months T1]). Changes in symbolic and nonsymbolic performance from Time 1 to Time 2 were positively correlated (r(28) = .57, *p* = .001, but not from Time 2 to Time 3 (r(28) = .32, *p* = .082).

The correlation between the changes in symbolic and nonsymbolic performance from Time 1 to Time 2 could be related to changes in nonsymbolic performance driving changes in symbolic performance, or vice-versa. To further investigate the nature of the relationship between symbolic and nonsymbolic performance at the beginning of Grade 1, we conducted two linear regressions to determine whether symbolic and nonsymbolic performance at Time 1 predicted Time 2 scores (See Tables 1 & 2). The first linear regression using Symbolic Time 2 scores as the dependent variable was significant, F(2,29) = 16.332, *p* < .001. In this model only symbolic Time 1 scores, but not nonsymbolic Time 1 scores, predicted unique variance in symbolic Time 2 scores. The second linear regression with nonsymbolic Time 2 scores as the dependent variable was significant, F(2,29) = 24.70, *p* < .001, and both symbolic and nonsymbolic scores at Time 1 predicted unique variance. Together, these results suggest that performance on the symbolic task influences nonsymbolic performance later in the year, however, nonsymbolic performance does not influence later symbolic performance. Consequently, the correlation between changes in symbolic and nonsymbolic performance from Time 1 to Time 2 may reflect how symbolic representations change nonsymbolic representations, rather than the reverse.

**Table 1:** Linear regression analysis predicting symbolic scores at Time 2 with symbolic and nonsymbolic scores at Time 1 as predictors.

Symbolic Time 2 Scores		
Predictor	β	t
Symbolic Time 1	.738**	**4.56**
Nonsymbolic Time 1	.005	**.024**

** *p* < .001

**Table 2:** Linear regression analysis predicting nonsymbolic scores at Time 2 with symbolic and nonsymbolic scores at Time 1 as predictors.

Nonsymbolic Time 2 Scores		
Predictor	β	t
Symbolic Time 1	.492*	3.44
Nonsymbolic Time 1	.406*	2.84

* *p* < .01

## Discussion

In the literature to date, it is unclear how symbolic skills develop and whether nonsymbolic representations of magnitude are the foundation on which number symbols are learned. Though several studies have examined changes in symbolic and nonsymbolic abilities cross-sectionally [13,31,35], few have examined these processes in the same sample of children [41]. In this study, we measured children’s symbolic and nonsymbolic skills longitudinally to address two main points: 1.) How symbolic and nonsymbolic skills develop over time and 2.) Whether the developmental trajectories of each format relate to one another. We found that children initially had better nonsymbolic skills, but performed equally well on symbolic and nonsymbolic number comparison by the end of the school year. The trajectories of the two formats (as measured by the slope of performance change) were not correlated with one another across the whole year. However, changes in symbolic and nonsymbolic performance were related to one another in the first half, but not the second half of the school year. This relationship was driven by symbolic representations predicting later nonsymbolic representations rather than the reverse. These findings suggest that by the first year of schooling, the development of symbolic skills is not dependent on earlier nonsymbolic representations. These findings further suggest that the relationship between nonsymbolic and symbolic magnitude processing is not unidirectional and that the acquisition of symbolic skills predicts the development of nonsymbolic skills.

### Developmental Trajectories of Symbolic and Nonsymbolic Processing

Despite the fact that little research has simultaneously examined symbolic and nonsymbolic processing abilities over time, cross-sectional data from Nosworthy and colleagues [35] has provided some insight into how symbolic magnitude representations change from Grade 1 to Grade 2. In particular, data from the present study and from Nosworthy et al. [35] indicate that Grade 1 is an important period for the development of symbolic magnitude processing skills where symbolic magnitude processing skills rapidly grow to match those of nonsymbolic processing skills. The present data replicate these findings and additionally show that symbolic magnitude processing skills have a distinct developmental trajectory from nonsymbolic magnitude processing skills in Grade 1, where symbolic skills improve at a faster rate than nonsymbolic skills. A study examining symbolic and nonsymbolic approximate arithmetic over a similar developmental window also demonstrated distinct developmental trajectories for symbolic and nonsymbolic abilities [41]. In this study, Xenidou-Dervou et al. [41] found that Kindergarten children did not show ratio-dependent performance on a symbolic approximate arithmetic task, however, by Grade 1 children demonstrated a canonical ratio effect. In contrast, there were no age related changes in the ratio effect for the nonsymbolic arithmetic task. Thus, the authors showed that symbolic approximate arithmetic abilities begin to develop in Grade 1, at the onset of formal math instruction. Using a different task (comparison instead of approximate arithmetic), our results are convergent with these findings by suggesting that symbolic and nonsymbolic magnitude processing abilities have distinct developmental trajectories. We further show that children have more rapid growth on symbolic compared to nonsymbolic skills in Grade 1. One possible reason for these different developmental trajectories might be related to differences in the role of domain general factors in the tasks. For example, nonsymbolic magnitude comparison tasks have been found to rely on inhibitory control [42–44]. Therefore, the development of nonsymbolic skills in the context of a magnitude comparison task may rely more strongly on the development of inhibitory control than symbolic magnitude processing. Future research will need to disentangle the role of these domain general factors in the development of magnitude processing skills.

It is possible that the developmental trajectories for symbolic and nonsymbolic processing may show more similarities earlier in development [16]. For example, children between the ages of 2–4 years may show more similar developmental trajectories when they are exposed to number symbols for the first time (for a discussion of mapping see the section below). The trajectories may also have different developmental timescales where nonsymbolic magnitude processing may begin earlier and progress more slowly than the development of symbolic skills. It is therefore possible that the present study has captured a developmental window where symbolic skills are growing at a much more rapid rate while nonsymbolic magnitude processing skills are developing more slowly. Together, these data suggest that by Grade 1 the symbolic and nonsymbolic abilities do not follow similar developmental trajectories, however, it is unclear whether this is the case for earlier developmental periods.

Following Grade 1, it is unclear whether the developmental trajectories for symbolic and nonsymbolic abilities continue to diverge or whether they begin to follow the same developmental trajectory. Previous research has found that adults do not share the same representations for symbolic and nonsymbolic magnitudes [30]. These data indicate the developmental trajectories may continue to diverge and stay distinct from one another into adulthood. It is also possible that the trajectories become more similar over time such that 2–4 year olds have two distinct systems for processing symbolic and nonsymbolic tasks, but by age 6, the systems become integrated into one unitary system [16]. However, we did not find such a pattern of results. Instead, our findings point to symbolic and nonsymbolic magnitudes having distinct representations as indicated by the dissociable developmental trajectories for symbolic and nonsymbolic processing. Based on our findings, and the results from Lyons et al. [30], we would predict that the developmental trajectories for symbolic and nonsymbolic abilities would continue to be distinct into adulthood.

### Symbolic Numbers and the Approximate Number System

How children learn the meaning of symbolic numbers is still an unanswered question. An ANS account proposes that nonsymbolic representations, or the ANS, is the foundation for symbolic number processing and culturally acquired mathematical skills (for example, see [10]). In contrast, our results show that symbolic and nonsymbolic skills are not as tightly linked as might be expected during a period that children are rapidly developing fluency with symbolic numbers. If symbolic and nonsymbolic formats had the same underlying representation in Grade 1, then we would expect that changes in one system would lead to changes in the other, however, we do not find such a relationship across the whole year. We do find that changes in performance in nonsymbolic performance relate to changes in symbolic performance in the first half of the school year, but that this relationship is predominantly driven by symbolic magnitude processing skills influencing later nonsymbolic performance. Consequently, the relationship between symbolic and nonsymbolic quantities may not be as unidirectional as previously thought [26,45].

The present findings also demonstrate a pattern of results that are not parsimonious with an ANS account. Namely, if the ANS is the foundation on which symbolic skills are learned, then we would predict a high correlation between the two formats from Time 1 because children who have a more precise ANS should also have better symbolic skills. Instead, we find that symbolic and nonsymbolic skills become more strongly correlated over time. This could indicate that children are developing a greater understanding of the relation between the formats (see discussion below), or that there are feedback effects such that symbolic skills influence ANS acuity [26] resulting in more similar symbolic and nonsymbolic skills over time. Indeed, we find evidence for such feedback effects within the first half of Grade 1, suggesting that the increasing similarity (particularly from the first to second time points) could be related to symbolic skills refining later nonsymbolic representations.

Other literature examining magnitude-processing skills at a similar age has reported comparable findings. For example, Sasanguie et al. [25] found that nonsymbolic performance in Kindergarten did not predict symbolic skills six months later. This suggests that the symbolic magnitude processing skills are not scaffolded onto nonsymbolic representations. Other longitudinal evidence has shown that symbolic skills in 3–4 year old children predicted nonsymbolic acuity 7 months later, however, the reverse was not found to be true [26]. In accordance with our findings, Mussolin and colleagues [26] provide evidence that symbolic and nonsymbolic knowledge influence each other in the opposite direction; symbolic skills influence the development of later nonsymbolic skills. The weak link between symbolic and nonsymbolic processing in children has also been illustrated in a recent study from Lyons et al. [27]. In a large sample of school-aged children, they found that a significant nonsymbolic ratio effect did not predict whether or not that child had a significant symbolic ratio effect. Together, these studies reveal a pattern of results that are not compatible with an ANS account, and show that symbolic magnitude representations are not necessarily grounded in nonsymbolic representations.

It should be noted, however, that not all available evidence points to different developmental trajectories for symbolic and nonsymbolic magnitude processing. In a study examining the magnitude processing skills from Kindergarten to Grade 1, Toll et al. [6] found a moderate relationship between the growth of symbolic and nonsymbolic skills, suggesting that the two formats influence each other. One explanation for the dissimilar results are the differences between tasks; the magnitude comparison task in the present study used digits from 1–9 whereas Toll et al. [6] used digits ranging from 0–100. It is possible that we did not have the power in our sample to detect a moderate, but significant, relationship between the developmental trajectories of the two formats. However, we did observe significantly different slopes between the symbolic and nonsymbolic formats, suggesting that the rate at which symbolic and nonsymbolic processing skills change over time is different.

It is possible that the relationship between symbolic and nonsymbolic representations is stronger prior to Grade 1. Children begin to have a conceptual understanding of symbolic numbers before the age of six. Therefore, we cannot discount the possibility that children’s initial experiences with symbolic numbers are tied to earlier nonsymbolic representations. However, our data support the notion that children’s increased fluency with symbolic numbers in Grade 1 is not tied to nonsymbolic abilities and instead, acquiring fluency with symbolic numbers may refine and modify nonsymbolic representations. As discussed above, other longitudinal research with younger children (3–5 years old) also challenges the prevailing view that symbolic representations are scaffolded on earlier nonsymbolic representations and converge with the present findings [25,26]. Consequently, several pieces of evidence converge to suggest that the development of symbolic numbers might not be as tightly linked to nonsymbolic representations as previously thought.

### Mapping between symbolic and nonsymbolic magnitudes

Our results do not provide evidence for the notion that symbolic numbers continue to be scaffolded onto nonsymbolic representations into the first year of formal schooling, however, mapping symbols onto their nonsymbolic quantities may still play an important role in children’s initial understanding symbolic numbers. For example, mapping between symbolic and nonsymbolic quantities has been shown to correlate with math performance [16–18], and may play a role in the transition from informal to formal math skills [46].

Though this study does not directly assess mapping abilities, it is possible that the increasing correlation between the two formats over time could be related to children acquiring better mapping skills over time. This increasing correlation between the two formats is also indicative of a changing relationship between symbolic and nonsymbolic quantities over time. Such correlations do not reveal a causal relationship between symbolic and nonsymbolic quantities. Consequently, nonsymbolic knowledge could influence the symbolic representations, symbolic knowledge could influence nonsymbolic representations [26], or the relationship could be bidirectional. In light of the current and previous evidence that demonstrates that symbolic number knowledge influences and refines the approximate number system [26], we suspect that the increasing correlations between the formats could be a byproduct of this effect. In other words, the development of symbolic number skills could be influencing the way in which nonsymbolic numbers are processed.

Another potential explanation for the increasing correlation between the formats over time could be the influence of domain general factors. It is possible that the development of general cognitive capacities, such as executive functioning, could facilitate performance on the task resulting in what appears to be a stronger relationship between symbolic and nonsymbolic performance over time. Consequently, future research will need to disentangle how domain general factors play a role in the development of magnitude processing skills and explore how the relationship between symbolic and nonsymbolic skills changes over developmental time.

### Limitations

Due to the nature of the design of the present study we cannot discount the possibility that test-retest effects could impact the rate of growth on the comparison task, and the possibility that the symbolic task could be more prone to test-retest improvements. However, we do not have a reason to believe that one format was more affected than the other because a separate investigation demonstrated that the test-retest reliability is similar across formats [36]. Consequently we suspect that differences in the rate of growth for the two formats are not solely related to differences in test-retest effects.

It is also worth nothing that any interpretations about symbolic and nonsymbolic representations are limited to children in Grade 1. As previously discussed, it is possible that symbolic and nonsymbolic representations are more closely linked earlier in development (however see [25] and [26] for research with younger children that demonstrate similar findings). Future longitudinal research will need to explore the developmental trajectories of symbolic and nonsymbolic abilities beginning earlier in development when children are first learning the meanings of number symbols.

## Conclusions

Understanding how magnitude processing skills develop is especially important because they have been shown to relate to arithmetic abilities in school children [2,4,47], particularly in the early school years [3]. Examining how symbolic magnitude processing skills develop can help elucidate how arithmetic becomes scaffolded on these basic numerical competencies over time. In the present study we have begun to uncover how symbolic and nonsymbolic skills develop in the first year of formal schooling, and how they relate to one another. We have demonstrated that symbolic magnitude processing skills show more rapid growth than nonsymbolic skills in Grade 1. We additionally find that the developmental trajectories of symbolic and nonsymbolic processing only relate to one another in the first half of the year and not across the whole year. However, we found that this relationship is driven by symbolic magnitude processing refining later nonsymbolic performance, contrary to what might be expected if symbols were grounded in nonsymbolic representations. Together, these findings suggest that nonsymbolic representations are no longer integral to the development of symbolic skills by the age of 6, and that the relationship between symbolic and nonsymbolic processing is not as unidirectional as previously thought. How children learn the meaning of number symbols remains an outstanding question. Some proposals have been put forth to suggest that symbols are first linked to exact nonsymbolic quantities in the subitizing range (digits 1–4) [48], and then the meaning of larger numbers are bootstrapped from an understanding of small numbers [49]. Given that there is not enough evidence to suggest that the ANS provides the foundation upon which symbolic numbers are learned, future research will need to explore how symbolic numbers are grounded and how children learn their meaning.
